# Ultra-fast sequence clustering from similarity networks with SiLiX

**DOI:** 10.1186/1471-2105-12-116

**Published:** 2011-04-22

**Authors:** Vincent Miele, Simon Penel, Laurent Duret

**Affiliations:** 1 Laboratoire Biométrie et Biologie Evolutive, Université de Lyon, Université Lyon 1, CNRS, INRIA, UMR5558; Villeurbanne, France

## Abstract

**Background:**

The number of gene sequences that are available for comparative genomics approaches is increasing extremely quickly. A current challenge is to be able to handle this huge amount of sequences in order to build families of homologous sequences in a reasonable time.

**Results:**

We present the software package SiLiX that implements a novel method which reconsiders single linkage clustering with a graph theoretical approach. A parallel version of the algorithms is also presented. As a demonstration of the ability of our software, we clustered more than 3 millions sequences from about 2 billion BLAST hits in 7 minutes, with a high clustering quality, both in terms of sensitivity and specificity.

**Conclusions:**

Comparing state-of-the-art software, SiLiX presents the best up-to-date capabilities to face the problem of clustering large collections of sequences. SiLiX is freely available at http://lbbe.univ-lyon1.fr/SiLiX.

## Background

Proteins can be naturally classified into families of homologous sequences that derive from a common ancestor. The comparison of homologous sequences and the analysis of their phylogenetic relationships provide very useful information regarding the structure, function and evolution of genes. Thanks to the progress of sequencing projects, this comparative approach can now be applied at the whole genome scale in many different taxa, and several databases have been developed to provide a simple access to collections of multiple sequence alignments and phylogenetic trees [1-9]. The building of such phylogenomic databases involves three steps that require important computing resources: 1) compare all proteins to each other to detect sequence similarities, 2) cluster homologous sequences into families (that we will call the *clustering *step) and 3) compute multiple sequence alignments and phylogenetic trees for each family. With the recent progress of sequencing technologies, there is an urgent need to *prepare for the deluge *and hence to develop methods able to deal with a huge quantity of sequences. In this paper, we present a new approach for the clustering of homologous sequences, based on single transitive links (*single linkage*) with alignment coverage constraints and implemented in a software package (called SiLiX for *SIngle LInkage Clustering of Sequences*). We model the dataset as a *similarity network *where sequences are vertices and similarities are edges [10]. To overcome memory limitations we follow an online framework [11] in which we visit the edges one at a time to update the families dynamically. This approach enables also an incremental procedure where sequences and similarities are added into the dataset so that it would not be necessary to rebuild the families from scratch. Finally, we adopt a divide-and-conquer strategy to deal with the quantity of data [12] and design a parallel algorithm whose theoretical complexity is addressed in this paper.

We evaluated the computational performances and scalability of this method on a very large dataset of more than 3 millions sequences from the HOGENOM phylogenomic database [9]. Our approach presents several advantages over other clustering algorithms: it is extremely fast, it requires only limited memory and can be run on a parallel architecture - which is essential for ensuring its scalability to large datasets. SiLiX outperforms other existing software programs both in terms of speed and memory requirements. Moreover, it allows a satisfying quality of clustering. We discuss the interest of SiLiX for the clustering of homologous sequences in huge datasets, possibly in combination with other clustering methods.

## Implementation

### Modelling

#### Single linkage and filtering with alignment coverage constraints

The principle of the single-linkage clustering is that if sequence A is considered homologous to sequence B, and B homologous to C, then A, B and C are grouped into the same family, whatever the level of similarity between A and C. The choice of the sequence similarity criteria that is used to infer homology is therefore an essential parameter of the single-linkage clustering approach. Different criteria can be used, separately or in combination (percentage of identity, alignment score or E-value, alignment coverage *i.e*. percentage of the length of the sequence that is effectively aligned). Then, if a pair of sequences (A, B) does not satisfy the criteria, the pair is not considered for the clustering. The choice of these criteria depends on the goal of the clustering.

**Figure 1:**
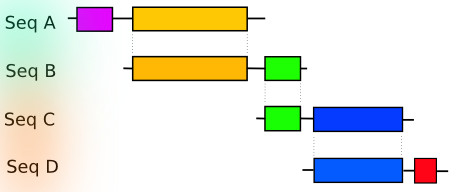
**Single linkage clustering with alignment coverage constraints**. The four proteins (A, B, C, D) contain some homologous domains (represented by colored boxes). To avoid the clustering in the same family of proteins that do not share any homology (e.g. A and D), pairwise sequence alignments are considered for the clustering only if they cover a minimum threshold of the length of each of the two proteins. This threshold has to be high enough to exclude cases like the alignment (B, C), which would lead to the clustering of A and D.

The method presented in this paper was motivated by the development of databases of homologous genes (such as HOGENOM or HOVERGEN [9]). The goal of these databases is to allow the study of the evolution of entire proteins considered as a unit, in contrast to databases such as PFAM [13] or PRODOM [14] that aim at studying the domain architecture of proteins. Hence, in HOGENOM, proteins are classified in the same family only if they are homologous over their entire length - or almost. In practice, protein sequences are compared against each other with BLASTP [15]. For each pairwise alignment, the list of High-scoring Segment Pairs (HSPs) is analyzed to exclude HSPs that are not compatible with a global alignment (for details, see [9]). Then, proteins are classified in the same family if the remaining HSPs cover at least a given *percentage of coverage *of the longest protein with a *percentage of identity *greater or equal to a given threshold (see Figure 1). Therefore the first step of the clustering process consists in analyzing pairwise sequence alignments resulting from the all-against-all comparisons (typically a set of alignments obtained with BLAST [15]) in order to obtain a binary information: keeping or excluding pairs whether they meet or not these sequence similarity criteria. This step (that we will refer to as the *filtering *step) can be time consuming, but can be easily distributed (see below).

#### Sequence families are the connected components of the similarity network

Here we consider the second step: given a list of pairs of similar sequences previously positively filtered, group the sequences into families. We define an undirected graph *G *= (*V*, *E*) with the set of vertices *V *representing sequences and the sets of edges *E *representing similarities between these sequences. We define *n *= |*V *| and *m *= |*E *|. Naturally, finding sequence families consists in computing the connected components of *G*. In this paper, we want to address the case of large *n *and *m *and we therefore develop a parsimonious approach in terms of memory use. We want to examine the edges *online *[11,16] and avoid storing them into a connectivity matrix. Therefore the classical *Depth-first search *algorithm [17] is not adapted. By analogy with external-memory graph algorithms [18], our approach consists in dynamically reducing the connected components into trees. When an edge is examined, we need to execute two operations: *find *the tree containing each of the two vertices and *union *these trees by merging their vertices into a new tree. Consequently, the connected component problem consists in (1) iteratively build a collection of trees representing the connected components of the graph *G *and (2) transform each resulting tree into a star tree which root is the representative (or *leader*) of the family. The final formulation of the problem is therefore building a spanning star forest G* = (*V*, E*).

### Using a memory-efficient structure

The connected components of *G *actually form a partition of *V *into non-overlapping subsets of vertices that we call *disjoint-sets*. Initially each vertex is a set by itself. We need to store the information of the partition and be able to update it dynamically. For this purpose, we use the *disjoint-sets data structure *[19,20] which is well suited when the graph is discovered edge by edge. This structure allows efficient implementation of the find and union operations by representing each set as a tree. Practically, the forest composed by all the trees is implemented as an array *parent *of size *n*. Each element *i *of a tree has a parent *parent*(*i*) such that *parent*(*r*) = *r *if *r *is the root of the tree. Moreover, it is straightforward and practical to transform each tree into a star tree such that the *parent *information is a common label for the vertices in a connected component. This will allow to directly retrieve each sequence family by reading the *parent *information.

#### Online procedure for a set of similarities

**Figure 2:**
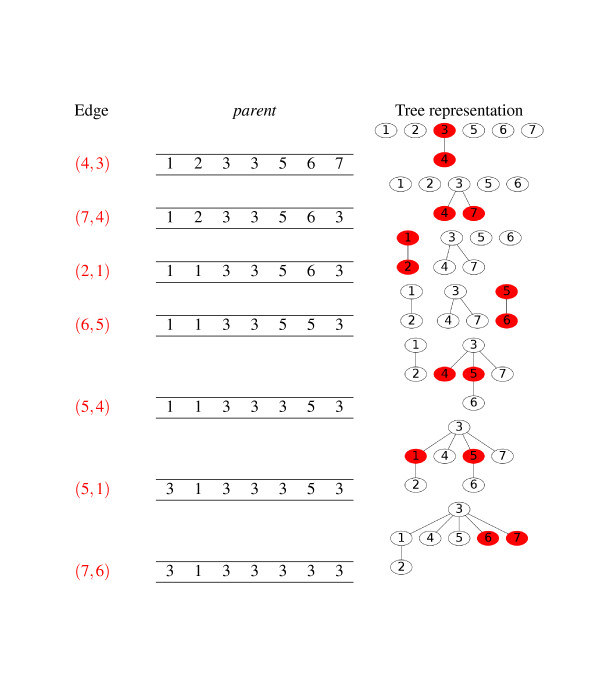
**An example of the steps involved in the algorithm called Union-Find by rank with path compression **[19,20]. Edges (first column, in red) are examined online. The disjoint-sets data structure, represented by trees (third column) and implemented using the *parent *array (second column), is consequently modified. The two vertices of the current edge of interest are colored in red.

To build G* from a set of sequence similarities, we develop a two steps procedure. First, we adopt the algorithm called *Union-Find by Rank with path compression *[19,20]. It consists in updating trees of minimal height while discovering the edges of the graph *G *online. For this purpose, the *rank *of a vertex is basically defined as its height in the tree. Each edge (*i*, *j*) is processed as explained in Algorithm 1 (see also Figure 2). It is basically based on the FIND function that associates the root of the tree containing a vertex of interest and the PATHCOMPRESSION function which connects the vertices in a path to the root of a tree. The time complexity was proved to be in our case almost *O*(*m*) [20]. Secondly, we use PATHCOMPRESSION for each vertex in *O*(*n*) time. This procedure requires the storage of *n parent *and *n rank *values such that the memory requirements are *O*(*n*).

**Algorithm 1 **ADDEDGE(*i*, *j*) by UNION- FIND

**Function: **FIND (*i*): returns the root of the tree containing *i*

**Function: **PATHCOMPRESSION(*i*, *r*): *parent *of vertices in the path from *i *to the root of the tree containing *i *are set to *r*

1: *r*_1 _← FIND(*i*); *r*_2 _← FIND(*j*)

2: *k *← arg max_*l *= 1, 2 _(*rank*(*r_l _*))

3: **if ***rank*(*r*_1_) == *rank*(*r*_2_) and *r*_1 _≠ *r*_2 _**then**

4:   *rank*(*r_k_*)++

5: **end if**

6: PATHCOMPRESSION(*i*, *r_k_*)

7: PATHCOMPRESSION(*j*, *r_k_*)

#### Parallelization for multiple sets of similarities

We take advantage of the possibility of exploring series of sets of sequence similarities with a client-server parallel architecture. We assume that it is usually affordable to split a large set into *q *sets. For the sake of clarity, we consider here a group of *q *processors, which is a reasonable hypothesis in practice. We note that it would also be recommended to have sets of comparable sizes. We adopt a divide-and-conquer strategy where different processors use the previous sequential algorithm to independently obtain a collection of spanning star forests  where  such that . These subsolutions are successively merged to obtain the final solution G* [12]. We first design an algorithm to merge two of these forests in *O*(*n*) time (see Algorithm 2). It is also based on the disjoint-sets data structure since, for each vertex *i*, it basically consists in adding in one forest a formal edge between *i *and the root of the tree containing *i *in the other forest. Then we build a parallel formulation of our approach [21,22] where  are obtained with step (1) of the sequential algorithm and iteratively merged (see Algorithm 3). The parallel time complexity can be estimated as *O*(*m*/*q *+ *nq*). We notice that the merge procedure is many orders of magnitude faster than the processing of a single set of similarities. For this reason, we decide not to distribute over the processors the merge procedures that will be consequently performed by the server processor in the order of the  availability.

**Algorithm 2 **MERGE

**Function: **FIND(*i*): returns the root of the tree containing *i*

1: **for all ***i *such that FIND(*i*) ≠ *i *in **do**

2:   r ← FIND(*i*) in 

3:   ADDEDGE(*r*, *i*) in 

4: **end for**

**Algorithm 3 **Parallel SiLiX

1: each processor *r *builds  with the sequential algorithm

2: **if ***r *> 1 client **then**

3:   MPI_SEND to server processor 1

4: **else**

5:   **for ***k *in 2,...*p ***do**

6:      MPI_RECEIVE among  in their order of availability

7:      MERGE

8:   **end for**

9:   **for all ***i *in **do**

10:      PATHCOMPRESSION(*i*, Find(*i*))

11:   **end for**

12: **end if**

### Dealing with partial sequences

#### Filtering

Because genome sequences are often not 100% complete and hence some genes may overlap with gaps in the genome assembly, it is important to be able to treat some *partial *protein sequences (as opposed to *complete *sequences). These partial sequences cannot be classified using the same criteria as the complete ones and are therefore treated separately. In a first step, gene families are built using only complete protein sequences as explained previously. In a second step, partial sequences are added to this classification, using different alignment length thresholds (for details about parameters, see [9]). It is important to note that, if there are several families that meet these alignment coverage criteria, a partial sequence is included in the one with which it shows the strongest similarity score.

#### Modelling

To allow the treatment of partial sequences, we propose a modified version of our approach. We redefine the previously mentioned graph *G *= (*V_c_*, *E_c_*) and we define the undirected graph *H *= *G *∪ (*V_p_*, *E_p_*) with two sets of vertices *V_c _*and *V_p_*, the complete and partial sequences respectively, and the set of edges *E_p _*between complete and partial sequences, each edge in *E_p _*being weighted by the similarity score. We also impose that edges between partial sequences are not allowed. In this case, *n_c _*= |*V_c_*|, *n_p _*= |*V_p_*|, *n *= *n_c _*+ *n_p_*, *m_c _*= |*E_c_*|, *m_p _*= |*E_p_*| and *m *= *m_c _*+ *m_p_*. At this point, we note that sequence families correspond to the connected components of a subgraph of *H *obtained by only conserving the edge of maximum weight for each vertex in *V_p_*: this will guarantee that each partial sequence is connected to only one complete sequence and prevent partial sequences to link two connected components. As described previously, the problem consists in building a novel graph  that has the following properties:

• H* is a spanning star forest,

• H* is called a *semi-bipartite graph, i.e*. a graph that can be partitioned into two exclusive and comprehensive parts (*V_c _*and *V_p_*) with internal edges (connecting vertices of the same part) only existing within one of the two parts [23]. The particularity is here that edges between the two parts are weighted,

• ∀*v *∈ *V_p_*, *deg*(*v*) = 1.

#### Online procedure and parallelization

First, it is necessary to insert an additional step between the two steps of the above-mentioned online procedure: build a subset of *E_p _*by selecting for each vertex the edge of maximal weight, in *O*(*m_p_*) time. Then we extend the step (2) to all the vertices in *V_p _*for a time complexity in *O*(*n*). This procedure runs in *O*(*n*) space since it requires the storage of *n parent *values. For the parallelized algorithm, we modify the merging of two forests presented in Algorithm 2 to consider vertices of *V_p _*and once again select edges of maximal weight, such that the overall parallel complexity can be estimated to be in *O*(*m*/*q*+ *nq*).

### The SiLiX software package

All the presented algorithms are implemented into the SiLiX software package which is written in ANSI C++ and uses MPI (Message Passing Interface) and elements of the well-established Boost library http://www.boost.org. SiLiX can take two kinds of input. First, the user can provide the result file of an all-against-all BLAST search (genomic or protein sequences) in tabular format (option -outfmt 6 in BLAST). In that case, SiLiX performs the filtering step by analyzing BLAST hits to search for pairs of sequences that fulfill similarity criteria (alignment coverage, sequence identity) set by the user to build families. In this mode, partial sequences can be treated separately, as described above. Second, if the user prefers to use other types of criteria for the filtering, SiLiX can simply take as input a list of pairs of sequences IDs and perform the clustering step. Compilation and installation are compliant with the GNU standard procedure. The package is freely available on the SiLiX webpage http://lbbe.univ-lyon1.fr/SiLiX. Online documentation and man pages are also available. SiLiX is licensed under the General Public License http://www.gnu.org/licenses/licenses.html.

## Results and Discussion

### SiLiX is faster and more memory efficient than other methods

To test SiLiX and compare it to state-of-the-art programs, we extracted protein sequences from the HOGENOM database (Release 5, [9]). The current release of HOGENOM contains 3,666,568 protein sequences (76% bacteria, 3% archae and 20% eukarya). We selected 3,159,593 non-redundant sequences including about 1% partial sequences. Sequences were compared against each others with BLASTP [15] with an E-value threshold set to 10^-4^. The BLAST output file contained 1,905,335,339 pairwise alignments. Then we selected three previously published programs, for which the source code is publicly available: *hcluster_sg *[24] and *MC*-*UPGMA *[25] that are based on hierarchical clustering, and *MCL *[26] that relies on graph-based heuristics.

The clustering of the protein dataset with SiLiX was very fast (about 2 hours) and required only limited memory capacity (0.4 GB). SiLiX outperformed the 3 other methods, both in terms of speed and RAM usage (see Table 1). The program *hcluster_sg *took 40 times more time than SiLiX to perform the clustering (about 4 days), and required a very large amount of RAM memory (99 GB). With larger sequence datasets (which are already present in databases), the RAM requirements of *hcluster_sg *will certainly exceed computer memory resources presently available. *MCL *also required a large amount of memory (78 GB) and was very slow (we stopped it after 10 days of calculation, before it finished the clustering). *MC-UPGMA *is almost as efficient as SiLiX in terms of RAM usage, but requires ample disk space to hold intermediate files (49 GB of HDD). The main problem with *MC-UPGMA *is that it is too slow on such a large dataset. *MC-UPGMA *uses an iterative procedure to cluster sequences. On our dataset, the first 20 iterations took 2 days. The authors of *MC-UPGMA *tested their method on a smaller dataset and they indicate in their article that 200 iterations were necessary to reach convergence (see [25]). We therefore extrapolated that *MC-UPGMA *would take more than 20 days on our dataset, and hence we decided to stop it before it finished the clustering. Note also that to optimize the performance of *MC-UPGMA*, the authors recommend using very permissive similarity threshold (E-value = 100, see [25]), which is unaffordable given the number of sequences in our dataset. In conclusion, SiLiX presents the best efficacy to tackle the challenge of huge dataset analysis with CPU and memory requirements equivalent to those of a laptop computer. Note that SiLiX may also be used in combination with other methods. To test this strategy, we first ran SiLiX with permissive similarity thresholds (sequence identity 25% and alignment coverage 80%), and then we used *hcluster_sg *to subdivide families of more than 100 sequences. This combined procedure still runs in a reasonable total CPU time (about 9 hours, i.e. 10 times faster than *hcluster_sg *alone, see Table 1) and also divides by 4 the RAM usage. Furthermore the second step of this combined procedure can easily be distributed on several computers.

**Table 1:** CPU time and memory requirements for SiLiX and three state-of-the-art programs on the dataset of similarity pairs extracted from the HOGENOM database [9].

method	CPU (min)	RAM (GB)
SiLiX	138	0.4
SiLiX + *hcluster_sg*^(100) ^[24]	552	23
*hcluster_sg *[24]	5604	99
*MC-UPGMA *[25]	>27000^(1)^	0.5
*MCL *[26]	>15000^(2)^	78
	^(1) ^estimated time (20 rounds took 2711 min., and 200 rounds were required in [25]) ^(2) ^manually stopped	

*hcluster_sg*^(100) ^corresponds to the use of *hcluster_sg *on the largest families (more than 100 sequences) retrieved by SiLiX with permissive similarity thresholds (sequence identity 25% and alignment coverage 80%). SiLiX and *MC-UPGMA *run on a quadri-quadcore Xeon 2.66 GHz with 24 GB RAM, *hcluster_sg *and *MCL *on a octo-quadcore Opteron 2.3 GHz with 64 GB RAM.

### SiLiX is scalable in practice

**Figure 3:**
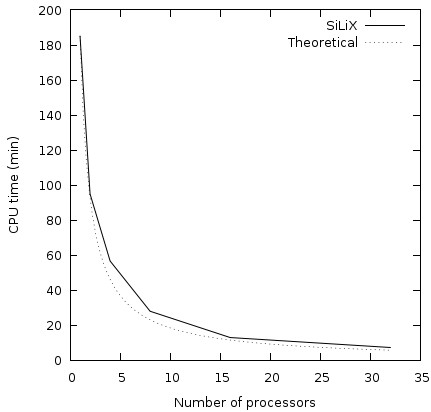
**CPU time of the parallelized version of **SiLiX**(plain) according to the number of processors on the dataset of similarity pairs extracted from the HOGENOM database **[9], **compared with theoretical values (dashed)**. Run on a cluster of 2 octo-bicore Opteron 2.8 Ghz and 2 octo-quadcore Opteron 2.3 GHz.

As the number of available sequences increases dramatically and the number of similarities is quadratic with this number of sequences, the CPU time required for the clustering is expected to increase very rapidly. To ensure the scalability of our method, we designed a parallel implementation of SiLiX with a low number of inter-processors communications to take advantage of multiple kinds of parallel hardware architectures. This algorithm delocalizes the processing of the sequence similarity dataset, including the filtering step, and merges the results in a last step (see Methods). We designed a divide-and-conquer approach that requires only *q *- 1 communications where *q *is the number of processors, with a procedure for merging partial results from two processors that is considerably faster than the independent computations on each processor. For these reasons, we observe practical performances consistent with the theoretical complexity such that the run time decrease is inversely-proportional to the number of processors (see Figure 3).

### Clustering quality

Although the speed and memory requirements are important parameters for the choice of clustering method, the most important criterion is of course the quality of the results. Single linkage clustering is known to be problematic because spurious similarities can lead to the clustering of non-homologous sequences. Even with stringent sequence similarity criteria, single linkage clustering can lead to erroneous clustering, because of the so-called problem of "domain chaining" [27], as illustrated in Figure 1. To avoid this problem, SiLiX performs single linkage clustering with alignment coverage constraints, i.e. pairs of similar sequences are considered for the clustering only if they meet two criteria: i) the alignment should cover at least a given percentage of the longest sequence; ii) sequence similarity within the alignment should exceed a given threshold. To assess the quality of SiLiX clustering, we used 2 different strategies. First, we compared clustering results to the classification of protein families reported in the InterPro database [28]. Second we assessed the performance of SiLiX on a set of 13 families of orthologous genes encoded by mitochondrial genomes in 1821 metazoan species.

#### Evaluation of SiLiX performances with Interpro

**Figure 4:**
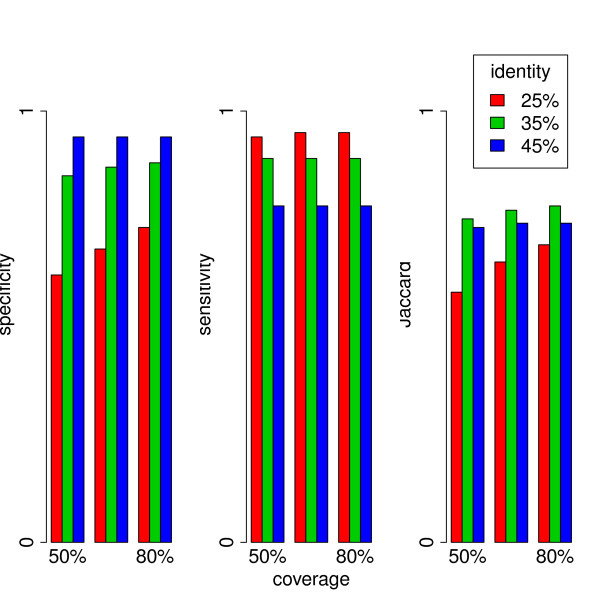
**Clustering performance evaluation based on InterPro classification**. a) specificity, b) sensitivity and c) Jaccard coefficient of SiLiX, used on similarity pairs extracted from the HOGENOM database, with different values of threshold on the percentage of sequence identity and alignment coverage.

We evaluated the performance of SiLiX on the HOGENOM dataset, using the procedure proposed by Loewenstein and colleagues [25]: we extracted the most frequent correspondence between SiLiX families and protein (not domain) families from InterPro (Release 22, [28]) containing more than 10 sequences, and then we calculated the specificity and sensitivity of SiLiX classification with respect to the InterPro family. We also computed the Jaccard score (see [25]), which is a standard metric of the trade-off between specificity and sensitivity. To evaluate the impact of sequence similarity criteria, we ran SiLiX with different thresholds for alignment coverage and percentage of identity. The performance of SiLiX were compared to those obtained with *hcluster_sg*, used alone or in combination with SiLiX. (NB: the 2 other methods, *MC-UPGMA *and *MCL*, could not be evaluated because of their excessive running time). As expected, increasing the alignment coverage and/or the sequence identity thresholds leads to increase specificity (Figure 4a), but decreases sensitivity (Figure 4b). The best trade-off between specificity and sensitivity was obtained for thresholds of 80% for alignment coverage and 35% for sequence identity (Figure 4c). With these parameters, the Jaccard score of SiLiX is slightly better than that of *hcluster_sg *(Table 2). Interestingly, the use of *hcluster_sg *in combination with SiLiX (with permissive threshold) leads to a better Jaccard score than *hcluster_sg *alone. Thus the use of SiLiX in combination with other methods can strongly decrease computing time without any loss in clustering quality.

**Table 2:** Comparison of clustering performances of SiLiX and *hcluster_sg *(used alone or in combination with SiLiX).

method (%identity)	**Jac**.	**Spec**.	**Sens**.
SiLiX (0.25)	0.69	0.73	**0.95**
SiLiX (0.35)	**0.78**	0.88	0.89
SiLiX (0.45)	0.74	**0.94**	0.78
SiLiX (0.25) + *hcluster_sg*^(100)^	0.77	0.85	0.92
*hcluster_sg*	0.76	0.84	0.91
			

Specificity, sensitivity and Jaccard coefficient were estimated by comparison with InterPro classification. The best quality metrics are indicated in bold. SiLiX was used with an alignment coverage threshold set to 80% and with various sequence identity thresholds. *hcluster_sg *was used on the entire dataset or only on the largest families (more than 100 sequences) retrieved by SiLiX with a permissive sequence identity thresholds of 25% (*hcluster_sg*^(100)^).

#### Evaluation of SiLiX performances with metazoan mitochondrial gene families

We used InterPro to evaluate clustering performances because this database is widely recognized for its quality and it has already been used for that purpose [25]. This strategy may however not be optimal because the building of the InterPro database also relies on arbitrary sequence similarity criteria. Hence, some cases that were considered as false positives in the above evaluations might in fact correspond to true homologues (i.e. specificity would be underestimated). Ideally, to evaluate clustering quality, one would need a set of homologous gene families known a priori, *i.e*. identified without using sequence similarity criteria. The mitochondrial genome of metazoan taxa can provide such an ideal test set. Indeed, in animals the mitochondrial genome contains 13 protein-coding genes. These proteins show different levels of sequence conservation across taxa, but the gene content is extremely conserved: except in very rare cases, all metazoan mitochondrial genomes contain these 13 genes [29,30]. This very strong conservation of synteny allows the identification of orthologous genes, even with very low levels of sequence similarity. We extracted from RefSeq (Release 41, [31]) a set of complete mitochondrial genomes from 1821 different species. The 13 mitochondrial proteins are present in all taxa, except ATP8 that is missing in the genome of 6 species. These mitochondrial proteins were then added to the HOGENOM dataset. Sequence comparisons were performed with BLASTP (using parameters indicated above), and the clustering was performed with SiLiX on the entire dataset, using thresholds of 80% for alignment coverage and 35% for sequence identity. For each of the 13 gene families, the majority of sequences were grouped in a single SiLiX family. The sensitivity, measured as the number of known proteins that are included in this first SiLiX family, is generally very high: it is higher than 94% for 11 out of the 13 gene families, and even higher than 99% for 9 of them (Table 3). The ND6 family was split into 3 main SiLiX families corresponding respectively to deuterostomia, protostomia and other metazoa (porifera, placozoa and cnidaria), and containing overall 95% of all known ND6 proteins. The ATP8 protein is very short (about 50 amino-acids) and evolves rapidly. The largest SiLiX family contains only 53% of all known ATP8, and the three largest SiLiX families contain 77% of known sequences, whereas 14% of known ATP8 were not included in any SiLiX family - in most cases because they did not have any BLAST hit with a E-value below 10^-4^. Thus, on this test set the sensitivity is generally very good, except for rapidly evolving sequences. To evaluate the specificity, we manually investigated all SiLiX families containing at least one protein of our mitochondrial set. We did not identify a single case where one mitochondrial protein was clustered with non-related proteins. Thus, even though the clustering was performed with the entire HOGENOM dataset (which contained more than 3 million different proteins), we did not detect any false positive clustering. This indicates that single linkage clustering with alignment coverage constraints is robust to spurious similarity matches.

**Table 3:** Evaluation of SiLiX performances on mitochondrial genes of metazoan taxa.

Gene	**Nb. Seq**.	**Nb**. SiLiX**families**	Nb. Seq. 1*^st ^*fam. (%)	Nb. Seq. 2*^nd ^*fam. (%)	Nb. Seq. 3*^rd ^*fam. (%)	Nb. Singletons (%)
ATP8	1815	26	959 (52.8)	294 (16.2)	144 (7.9)	258 (14.2)
ATP6	1821	2	1814 (99.6)	2 (0.1)	-	5 (0.3)
COX1	1821	1	1820 (99.9)	-	-	1 (0.1)
COX2	1821	1	1818 (99.8)	-	-	3 (0.2)
COX3	1821	1	1821 (100)	-	-	-
CYTB	1821	1	1820 (99.9)	-	-	1 (0.1)
ND1	1821	1	1821 (100)	-	-	-
ND2	1821	11	1714 (94.1)	53 (2.9)	3 (0.2)	34 (1.9)
ND3	1821	2	1813 (99.6)	2 (0.1)	-	6 (0.3)
ND4	1821	2	1812 (99.5)	2 (0.1)	-	7 (0.4)
ND4L	1821	7	1758 (96.5)	4 (0.2)	3 (0.2)	47 (2.6)
ND5	1821	2	1815 (99.7)	2 (0.1)	-	4 (0.2)
ND6	1821	16	1366 (75.0)	313 (17.2)	55 (3.0)	45 (2.5)

Protein-coding genes were extracted from complete mitochondrial genomes of 1821 different metazoan species, and clustered using SiLiX (see text). For each of the 13 genes, we indicate the number of sequences present in the dataset, the number of families (containing at least 2 sequences) identified by SiLiX that contained these sequences, and the number and percentage of sequences included in the 3 largest SiLiX families (when they exist), as well as the number and percentage of singletons (SiLiX families containing one single sequence).

## Conclusion

Different methods have been proposed for the clustering of proteins into families of homologous sequences [1,8,9,24-26,32]. These methods differ both in terms of the quality of the clustering, and in terms of the computing resources required to perform the clustering. The single-linkage clustering approach is used in different phylogenomic databases such as EnsemblCompara [8] or HOGENOM [9]. Here we propose a new implementation of the single linkage clustering method with alignment coverage constraints, SiLiX, which is extremely efficient, both in terms of computing time and memory requirements. Moreover, this method can be cost-effectively run on parallel architectures, and hence is easily scalable. Thus, in terms of the computing resource requirements, this method is much more efficient than other available methods for the treatment of huge sequence datasets. In terms of clustering quality, SiLiX performs as well as *hcluster_sg*, the only other available clustering program that could be run in reasonable time on such a large sequence dataset. Given its speed, SiLiX may also efficiently be used as a first clustering step, before running other algorithms.

## Availability and requirements

• Project name: SiLiX

• Project home page: http://lbbe.univ-lyon1.fr/SiLiX

• Operating system(s): All Unix-like operating systems such as Linux and Mac OS X.

• Programming language: C++

• Other requirements: MPI, the Boost:program options class, and optionally CppUnit and the Boost:unordered_map class.

• License: GNU GPL.

## Authors' contributions

VM developed the method and the software package. SP provided datasets and carried out the validation of the approach. LD initiated the work and participated in its coordination. All authors wrote the manuscript and gave final approval.
